# Grape Pomace as Innovative Flour for the Formulation of Functional Muffins: How Particle Size Affects the Nutritional, Textural and Sensory Properties

**DOI:** 10.3390/foods11121799

**Published:** 2022-06-18

**Authors:** Marica Troilo, Graziana Difonzo, Vito Michele Paradiso, Antonella Pasqualone, Francesco Caponio

**Affiliations:** 1 Department of Soil, Plant and Food Science (DISSPA), University of Bari Aldo Moro, Via Amendola, 165/a, I-70126 Bari, Italy; marica.troilo@uniba.it (M.T.); antonella.pasqualone@uniba.it (A.P.); francesco.caponio@uniba.it (F.C.); 2 Department of Biological and Environmental Sciences and Technologies, University of Salento, S.P. 6, Lecce-Monteroni, I-73100 Lecce, Italy; vito.paradiso@unisalento.it

**Keywords:** grape pomace, bakery products, dietary fiber, antioxidant compounds, winemaking process, by-products

## Abstract

Every year, the winemaking process generates large quantities of waste and by-products, the management of which is critical due to the large production in a limited period. Grape pomace is a source of bioactive compounds with antioxidant, anti-inflammatory, cardioprotective and antimicrobial properties. Its chemical composition makes it potentially suitable for preparing high-value food products. The aim of this research was to study the effect of adding grape pomace powder with different particle size fractions (600–425, 425–300, 300–212 and 212–150 µm) to the chemical, technological and sensorial characteristics of muffins. The addition of 15% of grape pomace powder, regardless of particle size, led to muffins rich in antioxidant compounds and total dietary fiber (>3/100 g), which could be labelled with the “source of fiber” nutritional claim according to the EC Regulation 1924/2006. As particle size decreased, total anthocyanins, total phenol content and antioxidant activity (evaluated by ABTS and DPPH assays) increased, while muffin hardness and lightness were negatively influenced. The latter observation was confirmed by the sensory evaluation, which also showed that a smaller particle size led to the presence of irregular crumb pores.

## 1. Introduction

Muffins are one of the most eaten bakery products in the world, highly appreciated both for their texture and taste and for ease of use and storage [1,2]. Their consumption is expected to grow globally. In fact, the global muffin market should reach almost 9 million dollars by the end of 2028, with a compound annual growth rate (CAGR) of about 2.6% during 2021–2028 [3]. However, muffins are high-calorie products with a low dietary fiber and protein content [4], not in line with the expectation of consumers for healthy foods. In this context, knowledge of the adverse effects associated with an unbalanced diet induced the development of functional foods and nutraceuticals [5].

Winemaking generates a large quantity of by-products, corresponding to 30% *w/w* of the starting grape used for wine production [6,7]. Grape pomace is the most abundant by-product, consisting of skin, pulp, residual stalks and seeds [8,9]. Traditionally, grape pomace is used to produce distillates, feeds and fertilizer [10]. There is a growing interest in the upcycling of food industry by-products, which is important to minimize the environmental impact and waste of resources from a circular economy perspective [11]. The chemical composition of grape pomace, characterized by its high concentration of phenolic compounds (phenolic acids, flavonoids, tannins and stilbenes), dietary fiber, proteins and minerals, makes it potentially suitable for preparing high-value food products [7,8]. The positive effects of these foods on human health, linked to the reduction in the risks associated with cardiovascular diseases, the prevention of diabetes and cancer, the reduction in LDL cholesterol and the prevention of obesity, are known [6].

The advantages linked to the presence of phenolic compounds of grape pomace are well known, both from a nutritional point of view, as well as from a technological point of view, for their antioxidant and antimicrobial action [8]. Gallic acid, trans-resveratrol, catechin and quercetin are involved in the prevention of cardiovascular diseases, cancer, osteoporosis and neurodegenerative diseases [12]. Moreover, the structure of phenolic compounds, the number and position of hydroxyl groups can influence their antimicrobial activity against bacteria, such as *Staphylococcus aureus*, *Escherichia coli*, *Streptococcus mutans* and *Listeria monocytogenes* [13].

Dietary fiber is defined as the edible parts of plants or analogous carbohydrates that are resistant to digestion and absorption in the human small intestine, with complete or partial fermentation in the large intestine [14]. Its beneficial effect is mainly linked to the reduction in the glycemic response and plasma cholesterol, as well as to its prebiotic effect; in addition, it improves the control of blood glucose in diabetic subjects, the intestinal immune response [9] and influences satiety, inhibiting intestinal digestive processes with a consequent reduction in the upper gastrointestinal transit time [15].

It is important to highlight that the daily dietary fiber intake recommended (25–30 g per day) [16] is almost never reached; food fortification could be a reasonable approach to increase dietary fiber intake. Many studies have shown the high content of dietary fiber in grape pomace, amounting to about 40–75% of the dry matter [2,17,18]. The inclusion of grape pomace in foods could allow one to obtain a functional muffin with beneficial effects related to both the presence of dietary fiber and polyphenols.

For this reason, in the last few years, several studies have been focused on the enrichment with grape pomace of different types of products, such as bread, biscuits and muffin [2,7,18,19,20,21,22].

With regard to muffins, Ortega-Heras et al. [2] found that the addition of different percentages of red and white grape pomace improved the nutritional characteristics, increasing dietary fiber and polyphenol contents, but worsened the sensorial and structural features (in terms of hardness and cohesion) when added at the highest percentages. These results confirmed the findings previously obtained by Bender et al. [20]. On the contrary, Nakov et al. [7] and Baldán et al. [19] highlighted an improvement in nutritional properties, as well as good results from the sensory point of view.

It is well known that the granulometry of flours has a great impact on the rheological properties of dough, as well as on the texture and sensorial characteristics of the obtained products [23].

The study of the particle sizes of grape pomace powders has been evaluated in previous studies in order to assess the impact of sieving on the physico-chemical (phenolic composition, antioxidant activity and microstructure) and functional properties of powders [24,25]. Moreover, in the literature, the effect of the different granulometry of grape pomace on the rheology of doughs is known, due to the significant influence on the viscoelasticity, gas-holding capacity and extensibility. The modulation of these parameters is essential for the quality of final products. Mironeasa et al. [26], in fact, highlight how the reduction in particle size of grape pomace powder affects the water-holding capacity, which is related to the chemical, textural and sensorial properties of enriched products.

Our work contributes to the evaluation of the effect of different particle size of grape pomace powders by assaying the nutritional, physico-chemical, textural and sensory features of the fortified muffins. In particular, four different particle size fractions (600–425, 425–300, 300–212 and 212–150 µm) were considered.

## 2. Materials and Methods

### 2.1. Chemicals and Reagents

Standards of gallic acid, syringic acid, quercetin-3-*O*-glucoside, (+)-catechin hydrate and myricetin were purchased from Sigma-Aldrich (St. Louis, MO, USA); *trans*-resveratrol was purchased from United States Pharmacopeia (USP) (Rockville, Canada); kaempferol, *ε*-viniferin, malvidin-3-glucoside, quercetin, rutin and catechin from Phyproof^®^ (Vestenbergsgreuth, Germany). Methanol HPLC grade and ethanol absolute anhydrous were purchased from Carlo Erba (Milan, Italy); acetonitrile HPLC grade and sodium carbonate from Honeywell (Seelze, Germany); formic acid, Folin–Ciocalteu reagent, ABTS (2,20-azino-bis (3-ethylbenzothiazoline-6-sulphonic acid) diammonium salt) and DPPH (2,2-diphenyl-1-picrylhydrazyl) were purchased from Sigma-Aldrich (St. Louis, MO, USA).

### 2.2. Grape Pomace Preparation

Red grape pomace (GP) (*Vitis vinifera* L., cultivar Primitivo) was kindly provided by a winery in Santeramo in Colle (Bari, Italy), following a maceration phase of seven days and collected after pressing. The red grape pomace was chosen in order to deal with the significant quantities of the by-product generated, linked to the large production of red wines in our territory. After preliminary tests at different drying temperatures and times, the grape pomace was dried at 120 °C for 60 min in a ventilated oven (Argolab-TCF120, Carpi, Italy) to obtain a moisture content of 3–4%, measured with a thermobalance (Radwag Mac 110/NP, Poland), as described by Difonzo et al. [27]. Then, the grape pomace was separated from the grape seeds by a 5 mm sieve (Endecotts test sieve, London, England), ground by an electric mill equipped with a sieve of 0.6 mm (ETA-Vercella, Turin, Italy) and sifted by different sieves (Giuliani Tecnologie srl, Turin, Italy) to obtain the following four different particle size fractions: 600–425, 425–300, 300–212 and 212–150 µm. The obtained powders had comparable protein, lipid, ash and total dietary fiber contents (13/100 g, 8/100 g, 9/100 g and 45/100 g on dry matter, respectively).

### 2.3. Muffin Preparation

**Figure 1:**
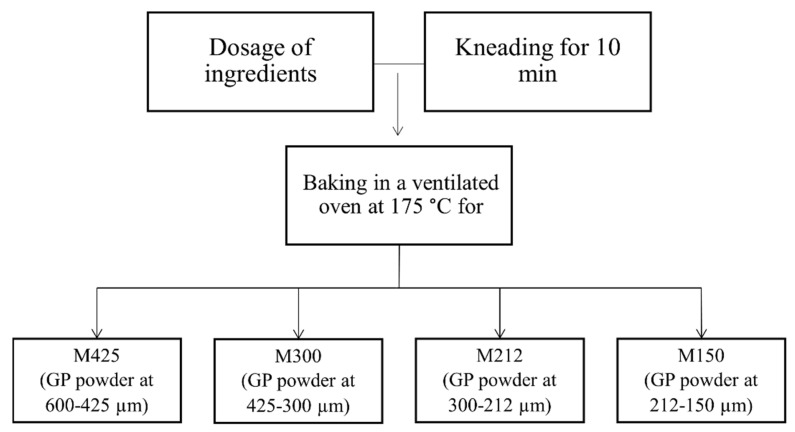
Muffin production process. GP: grape pomace.

Muffins were produced at Pasticceria Noviello (Altamura, Italy), according to the following formulation: 75 g of wheat flour 00 (Agugiaro & Figna Molini S.p.a., Collecchio, Italy), 15 g of grape pomace powder at different particle size, 125 g of eggs (Avicola Debernardis, Altamura, Italy), 33 g of milk (Latte Rugiada S.r.l, Matera, Italy), 5 g of baking powder (Agivega CSM, Crema, Italy), 67 g of sunflower seed oil (Olearia Desantis S.p.a., Bitonto, Italy), 40 g of potato starch (Südstarke GmbH, Schrobenhausen, Germany), 67 g of sugar (Mancinelli S.p.a., Viterbo, Italy) and 1.5 g of salt. The amount of 15/100 g of grape pomace powder was chosen to obtain muffins with an estimated total dietary fiber content of >3/100 g, which allows labelling with the “source of fiber” nutritional claim according to the EC Regulation 1924/2006 [28]. The production process is summarized in Figure 1.

The following four types of muffins were produced: M425 (grape pomace powder at 600–425 μm); M300 (grape pomace powder at 425–300 μm), M212 (grape pomace powder at 300–212 μm) and M150 (grape pomace powder at 212–150 μm).

### 2.4. Extraction and Determination of Phenolic Compounds

The phenolic compounds of grape pomace powders and muffins were extracted as described by Pintać et al. [29], with some modifications. Briefly, 1 g of sample was extracted with 10 mL of 80% methanol in an ultrasound bath (CEIA international S.A., 115/230 Vac 1- 50/60 Hz–400 VA max, Viciomaggio, Italy), for 15 min at room temperature, then shaken for 30 min and centrifuged (Thermo Fisher Scientific, Osterode am Harz, Germany) for 10 min at 8000× *g* at 4 °C. Three washes were performed for each sample.

The total phenol content (TPC) was determined according to the Folin–Ciocalteu method according to Difonzo et al. [30], with some modifications. To 20 µL of the filtered extracts, 980 µL of deionized water and 100 µL of Folin–Ciocalteu reagent were added. After 3 min, 800 µL of 7.5% Na_2_CO_3_ was added and then incubated at room temperature for 60 min. The absorbance was read at 720 nm using a Cary 60 spectrophotometer (Cernusco, Milan, Italy) and the TPC was expressed as mg of gallic acid equivalents (GAE)/g of the dry weight sample. Each sample was analyzed in triplicate.

The analysis of the phenolic profiles was carried out using an UHPLC Dionex Ultimate 3000 system (HPG 3200 RS binary pump, WPS-3000 TRS autosampler, TCC-3000 RS column oven and PDA, Thermo Fischer Scientific, Germering, Germany), following the method described by Pintać et al. [29], with some modifications. The separation of phenolic compounds was performed with an Acclaim™ 120 Å C18 column (3 µm particle size, 3 mm × 150 mm length, Thermo Fischer Scientific), maintained at 30 °C using a mobile phase consisting of (A) water/formic acid (90:10, *v/v*) and (B) acetonitrile/formic acid/water (59.9:0.1:40, *v/v/v*), at the constant flow rate of 0.709 mL/min. The gradient program solvent B was as follows: 0–1 min 2%; 1–5 min 12%; 5–13 min 30%; 13–21 min 100%; 21–28 min 100%; 28–32 min 2%. The total run time was 32 min with an injection volume of 20 µL; the UV absorbance was acquired at 280, 320 and 370 nm and the samples were injected in triplicate. Calibration was performed by injecting ten standards three times at seven different concentrations. Peak identification was performed by comparison of the retention times and diode array spectral characteristics with the external standards. These standards were as follows: two phenolic acids (gallic and syringic acids), five flavonols (quercetin, quercetin-3-O-glucoside, myricetin, rutin and kaempferol), one flavanol ((+)-catechin) and two stilbenes (resveratrol and ε-viniferin). Quantification of the phenolic compounds was expressed as mg/kg of the dry weight sample.

### 2.5. Extraction and Determination of Anthocyanins

Anthocyanin extraction from grape pomace powders and muffins was carried out according to Zhao et al. [31], with some modifications. Approximately, 1 g of sample was added to 10 mL of methanol/water/formic acid (80:18:2, *v/v/v*) and subjected to ultrasound treatment for 10 min and shacked for 30 min. Subsequently, the extracts were centrifuged at 4 °C at 8000× *g* for 10 min, the supernatants were separated and the pellets reprocessed three times, as described above.

Total anthocyanin content (TAC) was determined by UV–vis spectrophotometry, as described by Pasqualone et al. [32]. Briefly, the extracts were filtered with a 0.45 µm nylon filter and analyzed by reading the absorbance at 535 nm by a Cary 60 UV–vis spectrophotometer (Agilent Technologies). The results were expressed in mg cyanidin 3-*O*-glucoside/g of each sample and the determination were carried out in triplicate.

The quantification of the anthocyanin composition was performed using a UHPLC Dionex Ultimate 3000 system, following the method described by Tarricone et al. [33], with some variations. The HPLC conditions applied were the same as those described for the quantification of phenolic compounds. The mobile phase consisted of (A) water/formic acid (90:10, *v/v*) and (B) acetonitrile, at the constant flow rate of 0.709 mL/min. The gradient elution program of solvent B was as follows: 0–1 min 5%; 1–10 min 13%; 10–20 min 15%; 20–30 min 25%; 30–35 min 5%. The total run time was 35 min with an injection volume of 20 µL; detection was performed at 520 nm and quantitative analysis was performed according to the external standard method based on a calibration curve obtained by the injection of different concentrations of malvidin-3-glucoside. The results are expressed as mg of malvidin-3-glucoside equivalents per kg of each sample.

### 2.6. Antioxidant Activity Evaluation

The extracts were analyzed for the evaluation of antioxidant activity with DPPH and ABTS assays, as described by Difonzo et al. [27]. The DPPH assay was carried out by preparing a solution of DPPH 0.08 mM in ethanol. Then, in cuvettes for spectrophotometry, 50 µL of the sample was added to 950 µL of DPPH solution. After 30 min of incubation, the absorbance was read at 517 nm using a Cary 60 spectrophotometer. However, for the ABTS assay, an ABTS˙+ radical was generated by a reaction with potassium persulfate (K_2_S_2_O_8_), adding 25 mL of ABTS (7 mM in H_2_O) to 800 µL of K_2_S_2_O_8_ and incubated in the dark for 16 h. The reaction for evaluating the antioxidant activity was carried out in cuvettes for spectrophotometry, with 50 µL of each sample and 950 µL of ABTS˙^+^ solution. After 8 min, the absorbance was read at 734 nm. The results were expressed in µmol Trolox equivalents (TE)/g of dried pomace. Each sample was analyzed in triplicate.

### 2.7. Proximate Composition

The protein content (total nitrogen × 6.25) and ash content were determined according to the AOAC, 2006 [34] (methods 979.09 and 923.03, respectively), while the lipid content was determined by a Soxhlet apparatus, using diethyl ether as an extracting solvent (AOAC, methods 945.38 F, 2006). The determination of total dietary fiber was carried out by the enzymatic-gravimetric procedure as described in the AOAC (method 985.29, 2006). The carbohydrate content was determined as the difference subtracting the protein, ash, moisture and lipid contents from 100.

### 2.8. Textural Profile Analysis (TPA)

The textural profile analysis of each sample was carried out according to Dingeo et al. [35], with some modifications. The analysis was performed on muffin (2 × 2 × 2 cm) using a texture analyzer Z1.0 TN (Zwick Roell, Ulm, Germany), equipped with a stainless-steel cylindrical probe (36 mm diameter) and a 50 N load cell was used. Data were acquired by the TestXPertII version 3.41 software (Zwick Roell, Ulm, Germany). Two compressive cycles were performed at a 1 mm/s probe compression rate and 50% sample deformation in both the compression, with 5 s pauses before the second compression. The analyses were carried out on sixteen muffins (four per type, in triplicate).

### 2.9. Color and Image Analysis

The colorimetric determination was carried out on the crust and crumble of each muffin, using a colorimeter CM-600d (Konica Minolta, Tokyo, Japan) supported by the software Spectramagic NX (Konica Minolta, Tokyo, Japan). Lightness (*L* *), redness (*a* *, ±red-green) and yellowness (*b* *, ±yellow-blue) were determined as color coordinates. The parameters were measured at several points on both the crust and crumb.

The image analysis was performed according to Dingeo et al. [35], with some modifications. The muffins were cut horizontally and the images were acquired and elaborated through the ImageJ software (National Institutes of Health, Bethesda, Rockville, MD, USA). The images were converted into an 8-bit grayscale and cropped for a section of 30 × 30 mm. Subsequently, the cell resolution was improved by a thresholding function and the parameters were set to detect pores with an area >of 0.05 mm^2^ and included the pores’ area, mean area, circularity and solidity.

### 2.10. Specific Volume Determination

The volume of muffins was determined by rapeseed displacement as described in the AACC, 2000 [36] (method 10–05.01). Specific volume was calculated as the volume to weight ratio. Each sample was analyzed in triplicate.

### 2.11. Sensory Analysis

The sensory analysis of the muffins was carried out by a trained panel composed of nine judges, with ages ranging between 25 and 55 years, at the University of Bari Aldo Moro (Italy). All the judges had neither allergies nor food intolerances and were regular consumers of bakery products. The sensory analysis followed the ethical guidelines of the laboratory and written informed consent was obtained from each panelist. Muffins were evaluated using a linear unstructured scale of 10 cm, indicating the intensity of the selected attributes. Appearance aspects were evaluated, indicating the crust color intensity (0 = beige, 10 = dark brown), crumb color and crumb pore homogeneity (0 = inhomogeneous, 10 = homogeneous). The olfactometric descriptors instead evaluated the odor intensity (0 = odor different from that typical of commercial muffin, 10 = typical odor of commercial muffin) and the intensity of toasted, must, astringent and spicy hints; the intensity of sweet and acid were evaluated by tasting; in addition, the textural attributes perceived in the mouth in terms of softness and stickiness were assessed. Finally, the overall acceptability was evaluated.

### 2.12. Statistical Analysis

Analysis of variance (ANOVA) and Tukey’s test were carried out on the experimental data by Minitab Statistical Software (Minitab Inc., State College, PA, USA). The assumptions in terms of homogeneity of variance, independent residuals and normal distribution of residuals were guaranteed and differences were considered statistically significant at *p* < 0.05. The dataset has been subjected to Pearson’s correlation coefficient and analysis of the main components (PCA).

## 3. Results and Discussion

### 3.1. Characterization of the Grape Pomace Powders

Grape pomace powders of different particle sizes were characterized for phenolic content, as well as for antioxidant activity (Table 1). The TPC did not show statistical differences when varying the particle size, while the antioxidant activity, evaluated with ABTS and DPPH assays, showed lower values in coarser powders. The same trend was confirmed for total anthocyanin content, with lower values in the grape pomace powder of >425 µm, in line with the findings of Zhao et al. [24].

These differences could be due to the higher fragmentation of the structural components of grape pomace during the milling process, leading to a greater release of these components into thinner fractions [25].

The HPLC-DAD chromatographic analysis (Table 2) showed that the most represented phenolic compounds were gallic acid, catechin, rutin and quercetin-3-*O*-glucoside in all powders, regardless of the particle size, with concentrations between 400 and 770 mg/kg, comparable to those reported by Pintać et al. [29]. Regarding the effect of particle size, the phenolic compounds in particular flavanols, flavanols and stilbenes, were generally lower with the powder particle size of 150 µm, whereas the other powders showed negligible variations. The discrepancies between the content of total phenolic compounds determined by the Folin–Ciocalteu reagent and the results of the HPLC-DAD analysis could be due to the lack of selectivity of the Folin–Ciocalteu assay [37] and to the presence of compounds other than phenolic compounds that could cause interference absorbing at the same wavelength [38].

The anthocyanins’ profile determined by HPLC-DAD showed that the most abundant compounds were malvidin-3-glucoside and malvidin-3-coumaroyl-glucoside. Regarding powder particle sizes, contrary to the trend observed for phenolic acids, flavonols, flavanols and stilbenes, significantly higher amounts of anthocyanins were found in powders with the smallest particle size, probably due to the different degree of interaction with polysaccharides [39].

### 3.2. Characterization of Experimental Muffins

Table 3 reports the results of chemical composition, TPC and TAC, antioxidant activity and colorimetric indexes of the experimental muffins. On the whole, the chemical composition of the examined muffins was similar to those reported by other authors [2,7,20]. The moisture content did not show significant differences among the samples; lipid and ash contents were significantly higher in the muffins obtained with thinner grape pomace powders (M212 and M150). In fact, extensive reduction in particle size due to grinding could liberate more components due to cell breakage, which could explain the increased fat and ash contents detected; on the contrary, proteins and carbohydrates were generally significantly higher in the muffins made with a coarser powder (M425).

The total dietary fiber content was significantly higher in M300, although slight differences were found among the samples. In all cases, the incorporation of 15% of grape pomace powder allowed for the labelling of the the muffins as “source of fiber”, according to the Regulation (EC) 1924/2006. In fact, this claim is attributed to foods that contain at least 3 g of total dietary fiber per 100 g of product [28]. The benefit of the grape pomace dietary fiber enrichment was reported by Urquiaga et al. [40], who observed a reduction in fasting glycemia after four weeks of consumption of hamburgers.

Moreover, the muffins made with thinner powders (M150) showed slightly higher values of total polyphenols and total anthocyanin and, consequently, higher antioxidant activity. Similar polyphenols contents were reported by Pasqualone et al. [22] in fortified biscuits with grape pomace extracts and by Hayta et al. [41] in bread enriched with 5% of grape pomace powder.

Regarding color parameters, the crust showed significantly higher lightness (*L* *), redness (*a* *) and yellowness (*b* *) in M425. The values of color indexes generally significantly decreased with decreasing powder particle size. Similar findings were observed by Wang et al. [42] for pork meat gels added to soy okara with different particle sizes. Moreover, the values of lightness confirm the results reported by Nakov et al. [7] in cakes enriched with grape pomace. In addition, the crumb showed a significant decrease in the lightness of the muffins as the particle size decreased and, contrary to crust, significantly higher values of *a* * and *b* * were reported, probably due to the slightly higher anthocyanins content [2,43]. The different *L* * values of crust and crumb could be attributed to the Maillard reaction between reducing sugars, abundant in grape pomace and amino acids [7].

**Figure 2:**
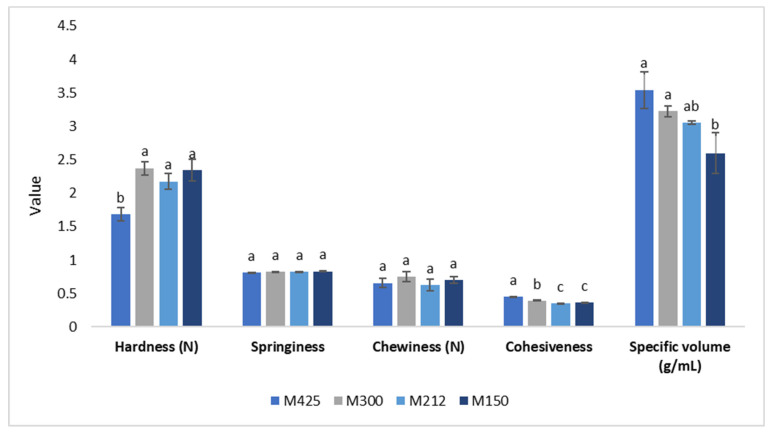
Mean values, standard deviation and results of statistical analysis (one-way ANOVA followed by Tukey’s HSD test for multiple comparison) of texture analysis and specific volume of experimental muffins. Different letters mean a significant difference at *p* < 0.05. M425, muffin with addition of grape pomace powder at 600–425 μm; M300, muffin with addition of grape pomace at 425–300 μm; M212, muffin with addition of grape pomace powder at 300–212 μm; M150, muffin with addition of grape pomace powder at 212–150 μm.

Figure 2 shows the results of the texture analysis (hardness, springiness, chewiness, cohesiveness) and the specific volume of the examined muffins. The results showed that varying the particle size of grape pomace powder, as a partial substitute of the flour, significantly affected hardness and cohesiveness, while springiness and chewiness showed no significant differences. In particular, the values of hardness and cohesiveness were inversely correlated and M425 showed significantly lower values of hardness and significantly higher values of cohesiveness. The data were in accordance with those found by Dhen et al. [44] in gluten-free cakes fortified with soy flour and Bressiani et al. [23] in wheat bread. These authors observed greater values of hardness when flours with smaller particle was used. The specific volume, calculated as the ratio of the volume occupied by the muffin to its weight, decreased as particle size decreased, with significant differences between the coarser and thinner particle sizes. This could be due to a different consistency and viscosity of the doughs that differently retained air bubbles and CO_2_, making the muffin more compact.

In contrast, Dhen et al. [44], following the addition of soy flour to gluten-free cakes, observed that the use of coarser particle sizes led to a decrease in specific volume, probably related to a decrease in cake height in the final stages of baking or cooling and to a coalescence of bubbles. In addition, de la Hera et al. [45] observed the same phenomenon as a consequence of the addition of rice flour with different particle sizes in the preparation of gluten-free rice-cakes, obtaining a higher specific volume when a thinner flour was used.

**Figure 3:**
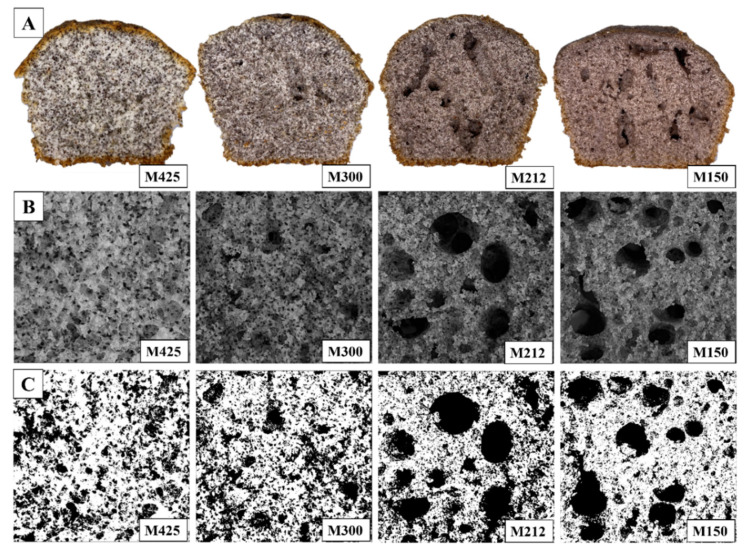
Pictures of transversal section of muffin prepared with different particle sizes: (**A**); longitudinal section of muffin crumb (**B**); binary images of muffin crumb (**C**). M425, muffin with addition of grape pomace powder at 600–425 μm; M300, muffin with addition of grape pomace at 425–300 μm; M212, muffin with addition of grape pomace powder at 300–212 μm; M150, muffin with addition of grape pomace powder at 212–150 μm.

The mean values related to cell area distribution, mean area, circularity and solidity of pores of muffin crumb are shown in Table 4. Muffins formulated with the smallest particle sizes (300–212 and 212–150 µm) showed significantly higher values of mean area, (1.07 and 1.13 mm^2^) than the muffins formulated with the coarsest particle sizes (*p* < 0.05). These variations could be related to the presence of larger pores, with areas in the range between 18 and 80 mm^2^, as reported in Table 4 and shown in Figure 3. Similarly, Sert et al. [46], following a high-pressure homogenization treatment, which led to a reduction in the particle size of the flours, showed an increase in the area of pores, with the increasing pressure used. Lazaridou et al. [47] and Qin et al. [48] have highlighted that the use of a coarser particle size of rice flour led to the formation of irregular and inhomogeneous air cell structures in the crumb of rusks and bread, respectively.

Circularity expresses the measure of the pore shape relative to a perfect circle (circularity = 1); while the solidity values (expressed as a ratio between the area and the convex area of the pore) equal to 1 indicate a smooth pore edge [49]. The muffins analyzed showed a trend directly proportional to the reduction in granulometry, indicating that the thinner powders used in the muffin formulation led to the development of less regular pores in shape, compared to the granulometry >300 µm. The solidity showed values inversely proportional to the particle size, indicating the presence of pores with smoother edges in the muffins made with the coarser powders. The opposite trend was found by Sert et al. [46], with solidity values increasing as the particle size of decreases. Therefore, it emerges that the reduction in the particle size of powder has induced a crumb with a heterogeneous porosity due to the presence of larger and irregular shape pores, as also found by Sert et al. [46].

**Figure 4:**
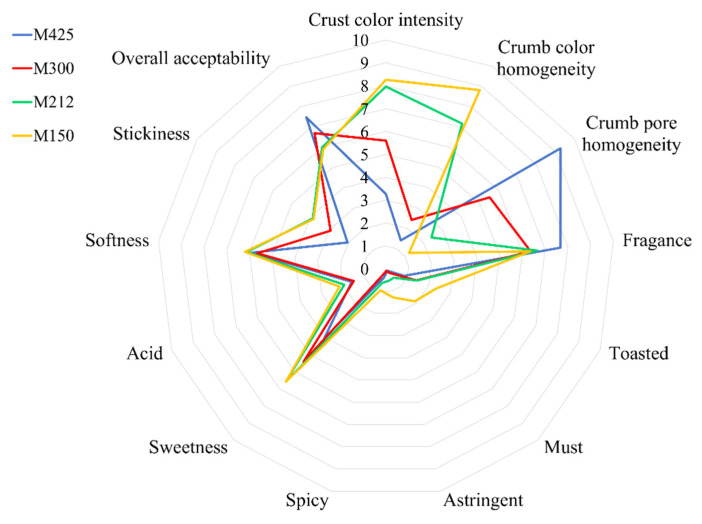
Results of the sensory analysis of the experimental muffins. Data are represented as means ± SD of nine replicates. M425, muffin with addition of grape pomace powder at 600–425 μm; M300, muffin with addition of grape pomace at 425–300 μm; M212, muffin with addition of grape pomace powder at 300–212 μm; M150, muffin with addition of grape pomace powder at 212–150 μm.

Figure 4 shows the results of the sensorial analysis of muffins. The obtained data highlighted that crust color was perceived to be significantly more intense in M212 and M150 (*p* < 0.05), confirming the results obtained by the instrumental analysis. The same trend was found for crumb color homogeneity, probably due to a more even mixing of the grape pomace powder with other ingredients. A similar result was observed by Majzoobi et al. [42] in gluten-free cakes enriched with carrot pomace powder of different particle sizes. The pore homogeneity, instead, was directly proportional to the granulometry; M425, in fact, was characterized by the presence of small and evenly distributed pores, compared to M212 and especially to M150, which had very irregular pores, as shown in Figure 3, confirming the results of the image analysis. No significant differences were reported in the intensity of the typical odor of the commercial muffins in the samples obtained with particle sizes lower than 300 µm, which differed from those prepared by adding coarser grape pomace powder. On the contrary, the perceptions of toasted, must, spicy and astringent, related to the presence of tannins in grape pomace, significantly increased with decreasing particle size (M150) (*p* < 0.05), presumably due to the lower content in dietary fiber, as evidenced by Torri et al. [50]. The presence of these perceptions was also reported by Kuchtová et al. [21] in cookies fortified with the powder of grape pomace and seeds; in particular, an astringent note was related to the interaction between the phenolic compounds and saliva in the mouth.

The perception of sweetness and acid showed increasing intensity as the particle size decreased. Finally, the texture attributes perceived in the mouth in terms of stickiness showed a significant increase in M212 and M150 (*p* < 0.05). Softness, instead, showed no differences among the samples, despite the fact that slightly higher values were observed in M212 and M150, with a score equal to 5–6 of 10. Acun and Gül [51] observed that the addition of more than 5% of red grape pomace in cookies led to an increase in hardness. On the whole, the overall acceptability showed a greater preference for muffins formulated with coarse particle size (M425 and M300), probably due to a lower impact on taste and smell in terms of acid, spicy and astringent, as well as for a more homogenous structure.

Table 5 summarizes the correlation coefficients (r) describing the degree of correlation among the measured chemical, textural and sensory parameters and particle size. The chemical parameters (antioxidant activity, TPC and TAC) showed a strong negative correlation (r > 0.5) with particle size, confirming the results reported previously (Table 3), according to which values decreased as the particle size increased. The positive but not significant correlation between TDF and granulometry should be noted, as well as the correlation with the colorimetric parameters in terms of L * of crust and crumb and in terms of a * and b * of crust. In contrast, the a * and b * of crumb showed a strong negative correlation with granulometry. However, from the textural point of view, the different particle size did not show a strong correlation with the parameters evaluated; while from the sensorial point of view, a strong negative correlation with the intensity of color of crust and with the alveolus homogeneity emerges, as well as with the hints of toasted, must, acid and spicy that increased as particle size decreased.

**Figure 5:**
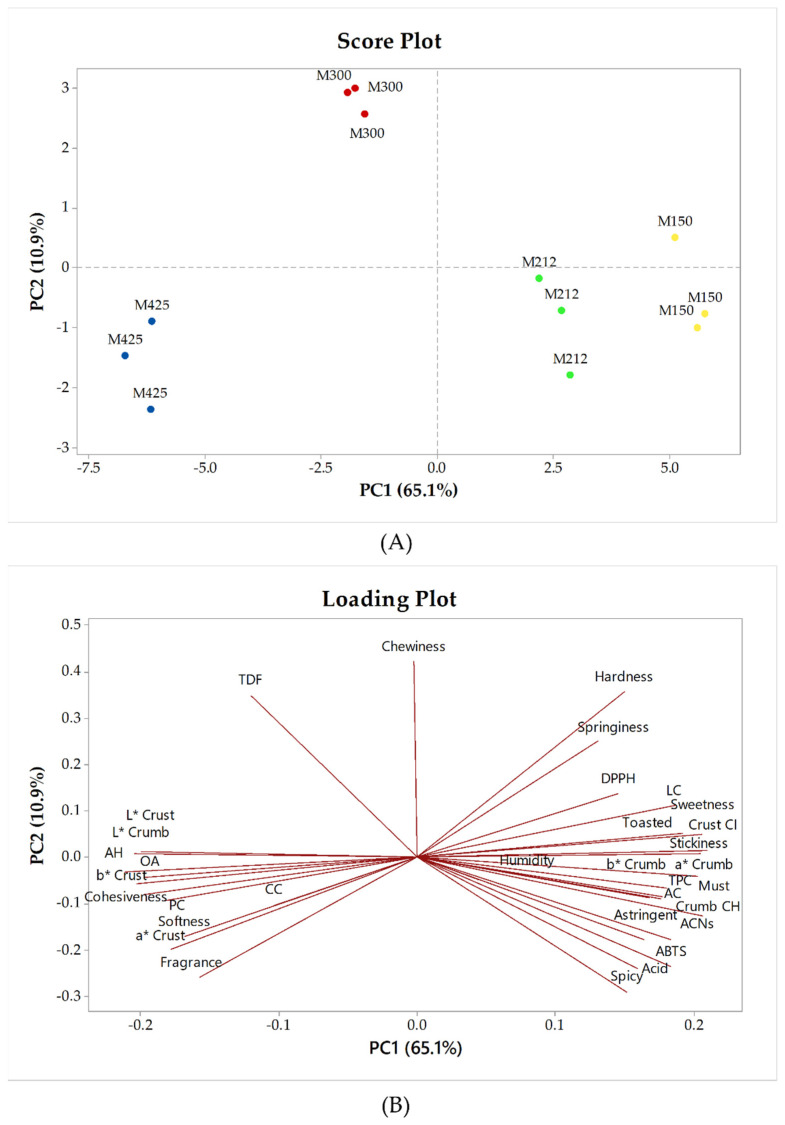
Score plot (**A**) and loading plot (**B**) of the principal component analysis (PCA) of experimental muffins. M425, muffin with addition of grape pomace powder with a particle size of 425 μm; M300, muffin with addition of grape pomace powder with a particle size of 300 μm; M212, muffin with addition of grape pomace powder with a particle size of 212 μm; M150, muffin with addition of grape pomace powder with a particle size of 150 μm. TDF: total dietary fiber; AH: alveolus homogeneity; OA: overall acceptability; PC: protein content; CC: carbohydrate content; LC: lipid content; crust CI: crust color intensity; TPC: total phenol content; AC: ash content; crumb CH: crumb color homogeneity; ACNs: anthocyanins.

In order to better evaluate the differences between the experimental muffins, the complete dataset was subjected to a principal component analysis (PCA). Figure 5 shows the score plot (Figure 5A) and the loading plot (Figure 5B) of the first two principal components. The first principal component (PC1) explained the 65.1% of the variance and discriminated the muffins with particle sizes of 425 and 300 µm (placed in the negative quadrants), from the muffins with thinner granulometry (M212 and M150), placed in the positive quadrant. In particular, M425 was discriminated based on the different colorimetric parameters (especially crust lightness), as well as for a greater pore homogeneity and odor intensity; in contrast, M150 was characterized by a higher phenolic content and antioxidant activity, by more intense acid, astringent, spicy and must hints and by more intense crust color and more homogeneous crumb color. Along the PC2 (10.9% of total variability), the muffins with particle sizes of 300 µm were separated from the others and plotted in the positive quadrants, characterized for slightly higher chewiness and total dietary fiber content.

## 4. Conclusions

This study explored the effect of the addition of grape pomace powder with different particle sizes on the chemical composition, textural and sensorial properties of enriched muffins. Particle size variation had an impact both on the anthocyanin content and antioxidant activity of powders, with increasing values as the particle size decreased; in contrast to the phenolic composition, which in terms of phenolic acids, flavonols, flavanols and stilbenes showed slight differences between the samples, with higher values at coarser particle sizes. The incorporation of 15% of grape pomace powder allowed the muffin to reach the total amount of dietary fiber of 3/100 g, allowing the muffins to be labelled as a “source of fiber”, as reported by EC Regulation 1924/2006 [28], especially for the coarser particle sizes analyzed. As expected, the muffins formulated with thinner granulometry showed higher values of antioxidant activity, anthocyanin and phenol content, although with small differences between the samples examined. However, from a textural and sensorial point of view, the smaller particle sizes negatively affected the hardness and color in terms of lightness, as well as the homogeneity of the pores. Overall, the proposed strategy allows one to upcycle the main wine-making by-product, obtaining high-value muffins. Future studies will be focused on the consumers’ acceptability to find a winning strategy to place the product on the market.

## Figures and Tables

**Table 1:** Mean values, standard deviation and results of statistical analysis (one-way ANOVA followed by Tukey’s HSD test for multiple comparison) of polyphenol and anthocyanin total contents and antioxidant activity of the different grape pomace size powder.

Parameters	600–425 µm	425–300 µm	300–212 µm	212–150 µm
Total phenols (mg/g)	10.39 ± 0.14 ^a^	12.39 ± 0.77 ^a^	11.69 ± 0.09 ^a^	11.18 ± 0.74 ^a^
Total anthocyanins (mg/g)	1.52 ± 0.02 ^c^	1.71 ± 0.03 ^b^	1.78 ± 0.01 ^a^	1.76 ± 0.01 ^a^
ABTS (µmol TE/g)	40.12 ± 1.46 ^b^	50.79 ± 2.10 ^a^	52.01 ± 2.25 ^a^	52.54 ± 1.69 ^a^
DPPH (µmol TE/g)	38.16 ± 0.90 ^b^	45.43 ± 0.77 ^a^	44.51 ± 0.24 ^a^	46.21 ± 0.52 ^a^

Data are represented as means ± SD of three replicates. Different letters in the same row mean a significant difference at *p* < 0.05.

**Table 2:** Mean values, standard deviation and results of statistical analysis (one-way ANOVA followed by Tukey’s HSD test for multiple comparison) of single phenolic and anthocyanin compounds determined by HPLC-DAD (expressed as mg/kg of dry matter) of the different grape pomace size powder.

Phenolic Profile	600–425 µm	425–300 µm	300–212 µm	212–150 µm
Phenolic acids				
Gallic acid	442.23 ± 3.23 ^c^	565.80 ± 4.29 ^a^	459.11 ± 7.20 ^b^	455.20 ± 3.90 ^b^
Syringic acid	205.71 ± 2.46 ^c^	217.60 ± 1.51 ^b^	237.65 ± 4.27 ^a^	176.29 ± 7.26 ^d^
Flavonols				
Quercetin	240.23 ± 22.23 ^a^	240.10 ± 0.62 ^a^	223.57 ± 2.73 ^b^	191.27 ± 2.22 ^c^
Quercetin-3-*O*-glucoside	485.60 ± 11.48 ^a^	474.96 ± 0.78 ^ab^	462.55 ± 4.22 ^b^	390.84 ± 2.38 ^c^
Myricetin	27.83 ± 0.92 ^a^	26.50 ± 0.32 ^a^	26.98 ± 0.74 ^a^	22.03 ± 0.78 ^b^
Rutin	485.29 ± 3.10 ^ab^	490.86 ± 2.46 ^a^	471.09 ± 9.90 ^b^	389.41 ± 3.61 ^c^
Kaempferol	39.89 ± 0.54 ^a^	37.25 ± 1.69 ^b^	36.31 ± 0.40 ^b^	28.06 ± 0.80 ^c^
Flavanols				
Catechin	785.19 ± 5.60 ^a^	770.64 ± 16.43 ^a^	767.55 ± 27.09 ^a^	718.20 ± 34.76 ^b^
Stilbenes				
*trans*-Resveratrol	27.47 ± 1.32 ^ab^	30.50 ± 1.20 ^a^	27.33 ± 1.19 ^b^	26.94 ± 1.01 ^b^
*ε*-Viniferin	32.00 ± 0.22 ^a^	29.38 ± 0.41 ^b^	25.12 ± 0.39 ^c^	19.17 ± 0.84 ^d^
Anthocyanins				
Delphinidin-3-glucoside	50.21 ± 0.89 ^c^	58.79 ± 0.84 ^a^	60.59 ± 1.44 ^a^	53.95 ± 1.89 ^b^
Cyanidin-3-glucoside	33.00 ± 1.59 ^c^	42.64 ± 1.73 ^a^	38.95 ± 0.51 ^b^	35.86 ± 1.45 ^bc^
Petunidin-3-glucoside	121.05 ± 2.86 ^b^	147.53 ± 1.55 ^a^	143.86 ± 3.15 ^a^	125.33 ± 0.35 ^b^
Peonidin-3-glucoside (mg/kg)	117.13 ± 4.18 ^b^	147.17 ± 3.34 ^a^	144.02 ± 6.23 ^a^	128.46 ± 5.02 ^b^
Malvidin-3-glucoside	811.68 ± 15.04 ^c^	975.32 ± 16.65 ^a^	952.16 ± 1.95 ^ab^	927.15 ± 9.05 ^b^
*cis*-Malvidin-3-coumaryl-glucoside	31.79 ± 1.03 ^b^	35.80 ± 1.20 ^a^	37.83 ± 1.37 ^a^	38.37 ± 1.33 ^a^
Malvidin-3-caffeoyl-glucoside	49.51 ± 0.28 ^c^	64.49 ± 1.79 ^b^	69.91 ± 3.01 ^ab^	73.44 ± 2.35 ^a^
Peonidin-3-coumaroyl-glucoside	128.26 ± 4.80 ^b^	179.08 ± 3.24 ^a^	186.07 ± 4.27 ^a^	188.59 ± 8.72 ^a^
Petunidin-3-coumaroyl-glucoside	95.66 ± 0.06 ^c^	121.85 ± 1.50 ^b^	133.09 ± 1.09 ^a^	135.00 ± 1.31 ^a^
*trans*-Malvidin-3-coumaroyl-glucoside	1789.30 ± 41.24 ^c^	2080.86 ± 40.96 ^b^	2175.09 ± 59.49 ^ab^	2278.61 ± 12.53 ^a^

Data are represented as means ± SD of three replicates. Different letters in the same row mean a significant difference at *p* < 0.05.

**Table 3:** Mean values, standard deviation and results of statistical analysis (one-way ANOVA followed by Tukey’s HSD test for multiple comparison) of proximate composition, polyphenol and anthocyanin total contents, antioxidant activity and crust and crumb colorimetric parameters (lightness, *L* *; redness, *a* *; yellowness, *b* *) of the different examined muffins.

Parameters	M425	M300	M212	M150
Moisture (g/100 g)	23.34 ± 2.70 ^a^	22.31 ± 0.54 ^a^	21.90 ± 0.58 ^a^	24.90 ± 1.07 ^a^
Lipids (g/100 g)	22.70 ± 0.57 ^b^	24.90 ± 0.77 ^a^	25.45 ± 0.73 ^a^	25.88 ± 0.71 ^a^
Proteins (g/100 g)	9.72 ± 0.26 ^a^	9.03 ± 0.17 ^b^	8.76 ± 0.14 ^b^	8.81 ± 0.10 ^b^
Ashes (g/100 g)	1.80 ± 0.18 ^b^	1.83 ± 0.05 ^b^	2.12 ± 0.05 ^a^	2.09 ± 0.01 ^a^
Carbohydrates (g/100 g)	40.04 ± 0.81 ^a^	38.01 ± 0.96 ^a^	38.67 ± 0.98 ^a^	35.26 ± 0.82 ^b^
Total dietary fiber (g/100 g)	3.40 ± 0.25 ^ab^	3.92 ± 0.24 ^a^	3.10 ± 0.21 ^b^	3.06 ± 0.16 ^b^
Total polyphenols (mg/g)	0.64 ± 0.01 ^b^	0.65 ± 0.01 ^ab^	0.68 ± 0.01 ^ab^	0.69 ± 0.01 ^a^
Total anthocyanins (µg/g)	25.20 ± 0.20 ^b^	24.50 ± 0.20 ^b^	28.03 ± 0.15 ^a^	28.03 ± 0.55 ^a^
ABTS (µmol TE/g)	2.23 ± 0.02 ^b^	2.21 ± 0.02 ^b^	2.50 ± 0.02 ^a^	2.49 ± 0.06 ^a^
DPPH (µmol TE/g)	1.72 ± 0.01 ^b^	1.75 ± 0.02 ^b^	1.74 ± 0.02 ^a^	1.78 ± 0.02 ^a^
Crust				
*L* *	43.85 ± 2.39 ^a^	40.82 ± 1.70 ^b^	37.76 ± 3.38 ^c^	36.73 ± 1.33 ^c^
*a* *	13.34 ± 1.42 ^a^	8.76 ± 2.23 ^b^	7.96 ± 1.74 ^b^	7.60 ± 2.09 ^b^
*b* *	26.81 ± 2.37 ^a^	20.90 ± 2.63 ^b^	17.10 ± 1.92 ^bc^	14.06 ± 3.96 ^c^
Crumb				
*L* *	61.64 ± 2.72 ^a^	54.60 ± 2.75 ^b^	44.18 ± 3.35 ^c^	46.34 ± 2.60 ^c^
*a* *	1.14 ± 0.15 ^d^	2.21 ± 0.25 ^c^	3.40 ± 0.36 ^b^	5.82 ± 0.30 ^a^
*b* *	9.17 ± 0.81 ^b^	9.50 ± 0.63 ^b^	9.90 ± 0.85 ^b^	11.53 ± 0.62 ^a^

M425, grape pomace powder at 600–425 μm; M300, grape pomace powder at 425–300 μm; M212, grape pomace powder at 300–212 μm; M150, grape pomace powder at 212–150 μm. Data are represented as means ± SD of three replicates. Different letters in the same row mean a significant difference at *p* < 0.05.

**Table 4:** Mean values and results of statistical analysis (one-way ANOVA followed by Tukey’s HSD test for multiple comparison) of cell area distribution, mean area, circularity and solidity of pores in the muffin crumb.

Range of Area (mm^2^)	Cell Area Distribution (%)			
	M425	M300	M212	M150
0.05–0.07	21.56 ± 0.93 ^c^	21.07 ± 0.76 ^c^	25.91 ± 0.62 ^a^	22.85 ± 0.03 ^b^
0.07–0.1	17.65 ± 0.27 ^b^	19.80 ± 1.79 ^b^	22.92 ± 0.54 ^a^	19.45 ± 0.66 ^b^
0.1–0.2	244.91 ± 0.62 ^a^	25.79 ± 0.28 ^a^	25.01 ± 1.06 ^a^	26.02 ± 0.84 ^a^
0.2–0.3	10.06 ± 0.37 ^a^	10.28 ± 0.06 ^a^	8.38 ± 0.48 ^b^	8.52 ± 0.38 ^b^
0.3–0.6	11.76 ± 0.36 ^a^	10.96 ± 0.65 ^ab^	7.66 ± 0.29 ^c^	9.94 ± 0.47 ^b^
0.6–2	10.50 ± 0.56 ^a^	8.35 ± 0.09 ^ab^	5.86 ± 0.17 ^c^	7.82 ± 0.66 ^ab^
2–8	3.55 ± 0.13 ^a^	3.52 ± 0.68 ^ab^	2.79 ± 0.26 ^b^	3.45 ± 0.61 ^ab^
8–18	-	0.23 ± 0.01 ^ab^	0.40 ± 0.03 ^a^	0.50 ± 0.09 ^a^
18–30	-	-	0.44 ± 0.06 ^a^	0.49 ± 0.04 ^a^
30–50	-	-	0.41 ± 0.06 ^a^	0.73 ± 0.09 ^a^
50–80	-	-	0.22 ± 0.03 ^a^	0.23 ± 0.01 ^a^
**Mean area** (**mm^2^**)	0.54 ± 0.01 ^b^	0.56 ± 0.13 ^b^	1.07 ± 0.15 ^a^	1.13 ± 0.10 ^a^
**Circularity**	0.47 ± 0.01 ^a^	0.47 ± 0.02 ^a^	0.37 ± 0.02 ^b^	0.34 ± 0.01 ^b^
**Solidity**	0.67 ± 0.01 ^a^	0.66 ± 0.01 ^a^	0.64 ± 0.01 ^b^	0.64 ± 0.01 ^b^

M425, grape pomace powder at 600–425 μm; M300, grape pomace powder at 425–300 μm; M212, grape pomace powder at 300–212 μm; M150, grape pomace powder at 212–150 μm. Data are represented as means ± SD of three replicates. Different letters in the same row mean a significant difference at *p* < 0.05.

**Table 5:** Pearson’s correlation coefficients (r) describing association of parameters evaluated and particle size.

Particle size	ABTS	DPPH	TPC	ACNs	TDF	L * Crust	a * Crust
	−0.998	−0.617	−0.969	−0.998	0.889	0.913	0.709
	b * Crust	L * Crumb	a * Crumb	b * Crumb	Hardness	Springiness	Chewiness
	0.869	0.927	−0.843	−0.761	−0.419	−0.482	0.429
	Cohesiveness	Crust CI	Crumb CH	AH	Fragrance	Toasted	Must
	0.849	−0.911	−0.976	0.894	0.482	−0.713	−0.728
	Sweetness	Acid	Spicy	Astringent	Softness	Stickiness	OA
	−0.884	−0.949	−0.863	−0.83	0.637	−0.907	0.988

Blue and red cells correspond to the positive and negative correlation coefficients (−1 and +1), respectively. TPC: total phenol content; ACNs: anthocyanins; TDF: total dietary fiber; crust CI: crust color intensity; crumb CH: crumb color homogeneity; AH: alveolus homogeneity; OA: overall acceptability.

## Data Availability

The data will be available on request.
